# Return-to-work of sick-listed workers without an employment contract – what works?

**DOI:** 10.1186/1471-2458-9-232

**Published:** 2009-07-14

**Authors:** Sylvia J Vermeulen, Sietske J Tamminga, Antonius JM Schellart, Jan Fekke Ybema, Johannes R Anema

**Affiliations:** 1 Department of Public and Occupational Health, EMGO Institute for Health and Care Research, VU University Medical Centre, Amsterdam, The Netherlands; 2 Dutch Research Center for Insurance Medicine AMC-UWV-VUmc, VU University Medical Center, Amsterdam, The Netherlands; 3 Coronel Institute of Occupational Health, Academic Medical Centre, University of Amsterdam, Amsterdam, The Netherlands; 4 TNO Quality of life, Hoofddorp, The Netherlands

## Abstract

**Background:**

In the past decade flexible labour market arrangements have emerged as a significant change in the European Union labour market. Studies suggest that these new types of labour arrangements may be linked to ill health, an increased risk for work disability, and inadequate vocational rehabilitation. Therefore, the objectives of this study were: 1. to examine demographic characteristics of workers without an employment contract sick-listed for at least 13 weeks, 2. to describe the content and frequency of occupational health care (OHC) interventions for these sick-listed workers, and 3. to examine OHC interventions as possible determinants for return-to-work (RTW) of these workers.

**Methods:**

A cohort of 1077 sick-listed workers without an employment contract were included at baseline, i.e. 13 weeks after reporting sick. Demographic variables were available at baseline. Measurement of cross-sectional data took place 4–6 months after inclusion. Primary outcome measures were: frequency of OHC interventions and RTW-rates. Measured confounding variables were: gender, age, type of worker (temporary agency worker, unemployed worker, or remaining worker without employment contract), level of education, reason for absenteeism (diagnosis), and perceived health. The association between OHC interventions and RTW was analysed with a logistic multiple regression analysis.

**Results:**

At 7–9 months after the first day of reporting sick only 19% of the workers had (partially or completely) returned to work, and most workers perceived their health as fairly poor or poor. The most frequently reported (49%) intervention was 'the OHC professional discussed RTW'. However, the intervention 'OHC professional made and discussed a RTW action plan' was reported by only 19% of the respondents. The logistic multiple regression analysis showed a significant positive association between RTW and the interventions: 'OHC professional discussed RTW'; and 'OHC professional made and discussed a RTW action plan'. The intervention 'OHC professional referred sick-listed worker to a vocational rehabilitation agency' was significantly associated with no RTW.

**Conclusion:**

This is the first time that characteristics of a large cohort of sick-listed workers without an employment contract were examined. An experimental or prospective study is needed to explore the causal nature of the associations found between OHC interventions and RTW.

## Background

### New types of labour market arrangements and work disability

In the past decade flexible labour market arrangements have emerged as a significant change in the European Union labour market. As a result the standard form of production, i.e. employees with a fulltime permanent and regular job, has made way to an upcoming of flexible workers, such as fixed-term employees and workers without an employment contract [1-4]. Workers without an employment contract are for instance temporary agency workers and unemployed workers. Studies suggest that these new types of labour arrangements may be linked to ill health[1,3-10] and an increased risk for work disability[2,4,11]. In the Netherlands, this is reflected in the absenteeism pattern, which is characterised by a higher annual sick leave rate for workers without an employment contract compared to employees (2004; 8,3% temporary agency workers, 6,3% national mean)[12,13], and a lower outflow in the first year of sickness absence with a higher inflow into a long term disability pension after one year compared to employees (2004; 1,1% temporary agency workers, 0.76% national level)[14]. It is stated that one of the causes is a greater distance to the labour market due to a larger proportion of workers with lower credentials, lower income, more females, more (partly) occupationally disabled, and more immigrants[2,13,15]. Another cause could be that occupational health care (OHC) and return-to-work (RTW) guidance for workers without an employment contract are inadequate[13].

### The Dutch Social Security System

There are many countries where sick-listing can only occur when an individual is gainfully employed. However, in the Netherlands the Sickness Benefits Act provides also for workers without an employment contract who become sick-listed. These workers, i.e. unemployed workers and temporary agency workers, can apply for a sickness benefit at the Social Security Agency (SSA) and receive 70% of their last daily wage during the first two years of sickness absence. In the absence of an employment contract there are no legislative mandates for these workers to be returned to their previous/last job. Therefore, the SSA is also responsible for OHC, i.e. sickness absence counselling and vocational rehabilitation of sick-listed workers without an employment contract. The sickness absence counselling is done by an insurance physician. The vocational rehabilitation is carried out by a team of OHC professionals, consisting of the insurance physician, a labour expert and a case-manager.

To claim sickness benefit, the sick-listed worker is obligated to report sick within two days after the start of sickness absence. He/she then automatically becomes entitled to OHC by the SSA for the duration of the sickness benefit. Based on the cause of sickness absence, i.e. diagnosis, the insurance physician of the SSA guides the worker according to the accompanying Dutch guideline for OHC, formulated by the Dutch association of occupational physicians. In addition, there are general obligatory OHC actions as dictated by Dutch legislation, i.e. the Improved Gatekeeper Law. For instance, summoning to consulting hours, discussing RTW with the sick-listed worker, and advising about actual starting with work again. The visits to the SSA are not voluntary. Not visiting the OHC professional and/or not cooperating with regard to recovery and RTW is punished, i.e. payment of the sickness benefit is stopped. When clients are 13 weeks sick-listed they have been invited to visit the insurance physician of the SSA at least once. The aim of this first medical assessment is dual, namely to certify sickness and thereby approving the sickness benefit claim, and a to make a (medical) problem analysis with advising about recovery, i.e. health promotion, and RTW possibilities. The insurance physician is not responsible for treating illness. This medical role belongs to the clients' general practitioner and/or other involved medical specialists. However, the insurance physician can advise and refer to work disability oriented treatment/guidance, for instance graded physical therapy or work-related psychological help. The OHC by the SSA ends when the worker is no longer sick-listed and the sickness benefit ends. This moving from being sick-listed to 'recovery' can be initiated by either the client or the insurance physician. The client can report being recovered from illness and/or starting with work again, i.e. full RTW. The insurance physician can establish full recovery of health and/or full work ability (with or without actual RTW of the client). When the worker is still partially or fully work disabled after two years, he/she can apply for a long-term disability benefit. This is the same as for long-term sick-listed employees.

### Flexible labour market arrangements: the temporary agency worker

Temporary agency work is a form of a flexible labour market arrangement. There is a triangular relationship (as opposed to the bilateral relationship between an employer and employee) between the worker, a company acting as a temporary work agency, and a user company. The temporary work agency places the worker at the disposition of the user company and the work is of temporary nature without a labour agreement. This in contrast to a temporary worker with a fixed-term contract. In the Netherlands, temporary workers with a fixed-term contract are viewed as employees with legislative responsibilities for the employer regarding payment of the daily wage and RTW guidance when the fixed-term employee becomes sick-listed.

### Objectives

To date, only a few studies have been conducted with regard to OHC and RTW of the group of sick-listed workers without an employment contract. Therefore, the objectives of this study were: 1. to examine demographic characteristics of workers without an employment contract who are sick-listed for at least 13 weeks, 2. to describe the content and frequency of OHC interventions by the insurance physician of the SSA for these sick-listed workers without an employment contract, and 3. to examine the association between applied OHC interventions and RTW for sick-listed workers without an employment contract, accounting for possible confounding variables.

## Methods

### Cohort recruitment and data collection

**Figure 1:**
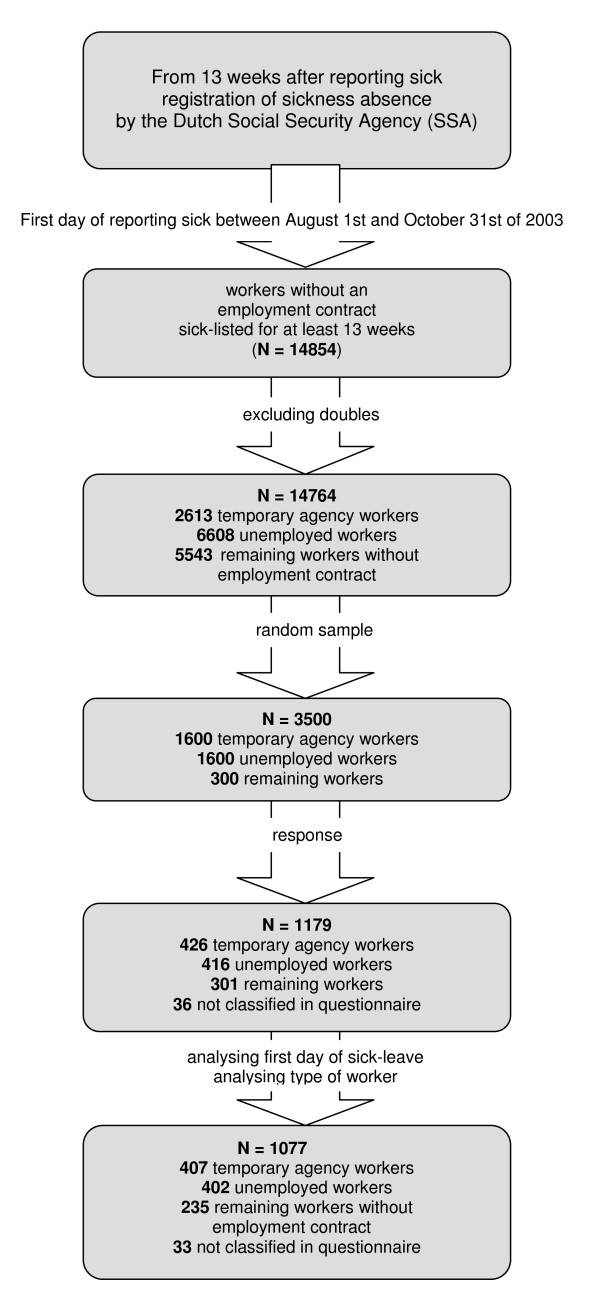
**Cohort recruitment**. Summary of the cohort recruitment of workers without an employment contract, sick-listed for at least 13 weeks.

This study was part of a series of Dutch researches regarding OHC and RTW among employees and workers without an employment contract[16]. The study was commissioned by the Dutch Ministry of Social Affairs and Employment and conducted by the Netherlands Organisation for Applied Scientific Research (TNO) from May 2004 until June 2004. Inclusion criteria for the study population in this cohort study were: workers without an employment contract, who had reported sick between the first of August and the end of October of 2003 and who were at baseline at least 13 weeks sick-listed[16]. This 13 week period related to the registration of sickness absence by the Dutch Social Security Agency (SSA), which started 13 weeks after the first day of reporting sick. A sample of 3.500 persons was random drawn by the SSA from a total population of 14.854 workers without an employment contract, who had reported sick between the first of August and the end of October of 2003 and were at baseline at least 13 weeks sick-listed[16]. Using the available data of the population, a non-response analysis was conducted to look at the possibility of selectivity of the response (n = 1077). Next, based on the registration by the SSA, the sample was then divided into the following three representative subgroups: temporary agency workers, unemployed workers, and remaining workers. This latter subgroup consisted for instance of people who had partly a disability pension and worked partly as a temporary agency worker. Only demographic variables were available at baseline. Measurement took place 7–9 months after the first day of reporting sick, i.e. 4–6 months after inclusion. A questionnaire was send to the study population by mail by the SSA in May 2004 and after one month a written reminder was sent to the study population who had not returned the questionnaire. Due to privacy considerations it was not possible to call the respondents if the received questionnaires were not complete or if there was anything unclear. In total 1179 questionnaires (response rate of 34%) were received. The three subgroups were then redivided based on the type of worker as reported by the clients. Next, after analysing the reported first day of sick leave (56 of the 1179 respondents had a first day of sickness absence which did not fall between the first of August 2003 and the end of October 2003), and analysing the type of worker (i.e. respondents with a full disability pension or an employment contract were excluded), the remaining group consisted of 407 temporary agency workers, 402 unemployed workers, 235 remaining workers without an employment contract, and 33 workers not classified (unknown). In conclusion, the cohort in this study consisted of 1077 workers without an employment contract. The cohort recruitment is summarised in figure 1.

### Questionnaire

**Figure 2:**
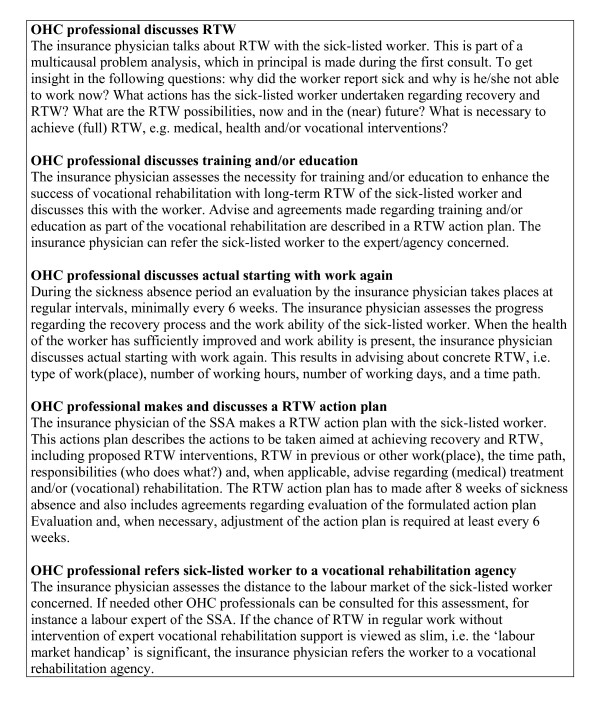
**Overview of examined occupational health care interventions**.

The self-reported questionnaire was developed by the Netherlands Organisation for Applied Scientific Research (TNO) and modelled after a questionnaire to examine OHC among employees, which was used four years earlier[17]. The first part of the questionnaire gave information about RTW (full RTW was defined as working in any type of job, i.e. work with or without a contract, and the number of working hours same as the last work before reporting sick), first date of sick leave, cause of absenteeism (health complaint), perceived health, and employment status. The second part gave information about OHC interventions carried out by the insurance physician of the SSA. These questions related to obligatory interventions, which were required according to Dutch legislation for OHC, i.e. the Improved Gatekeeper Act (for an overview of the examined OHC interventions see figure 2). Questions about the received OHC interventions were answered with 'yes', 'no' or 'do not know'. In the last part demographic characteristics were asked, such as age, gender, and level of education.

### Statistical methods

Most data in this study were of a descriptive nature. All variables were on a binominal or categorical level. Numbers and percentages were rounded to the nearest point. Next, a model was built with logistic multiple regression (listwise) to identify which OHC interventions were determinants for RTW, accounting for possible confounding variables and interaction effects. In the first step, the possible determinants were selected one by one for significance. Next, possible confounders were added to the model one by one. If a possible confounder altered the beta coefficient of one of the selected determinants with 10% or more, this confounder entered the model. For the selected determinants significance level was reached when the p-value was ≤ 0.05. In the last step, the possible interactions between the confounders and the selected determinants were examined. If relevant interactions were significant these were added to the end model. Before conducting the logistic multiple regression analysis the bi-variate (Spearman) correlations of all the involved independent variables were checked to see whether or not problems due to multicollinearity could arise. All analyses were performed using the SPSS 15.0 software package (SPSS Inc., Illinois, USA).

### Modification of variables

Two variables were modified before analysing. The first variable was the way in which the respondents had returned to work. They could choose from the following options: not returned to work, returned to work on a therapeutic basis (partially or complete), partially returned to work, and completely returned to work. For analysing the RTW-rates, due to the small numbers of therapeutic return-to-work, the variable was first converted into the following values: not returned to work, partially returned to work (this included partial or complete therapeutic return-to-work), or completely returned to work. Then, for the logistic multiple regression analysis RTW was modified into a binominal variable, i.e. returned to work (partially or completely) and not returned to work. The second variable which was converted was the reason for absenteeism, because a lot of the respondents filled in the category 'remaining complaints' instead of the categories cardio-vascular disease, mental health complaints, or musculoskeletal complaints. When the health complaints were described or clarified in the category remaining complaints, if possible, the diagnosis was manually reclassified by the researchers into one of the above mentioned categories.

## Results

### Baseline characteristics of the cohort

In table 1 the results, i.e. frequencies, are presented for gender, age, type of worker, and level of education. Men and women were equally represented in this cohort study (49% versus 51%). The mean age was approximately 41 years with 75% of the workers equally distributed in the range between 25 and 54 years. Comparing the bottom age range (15–25 years) with the top age range (≥ 55 years) showed that the cohort consisted of more older workers. The youngest workers were with only 9% the smallest category. When looking at the level of education, more than half of the workers had a low level education. Only 14% of the workers had a high level education.

**Table 1:** Baseline demographic characteristics of the cohort of workers without an employment contract (n = 1077)

**Demographic characteristics**		**Cohort (n = 1077)**
**Gender**	*Woman*	51%
	*Man*	49%

		

**Age**	*15–24 year*	9%
	*25–34 year*	23%
	*35–44 year*	28%
	*45–54 year*	25%
	≥ *55 year*	15%
	*Mean (sd) age (years)*	41.1 (11.4)

		

**Level of Education**	*Low*	54%
	*Average*	32%
	*High*	14%

		

**Type of worker**	*Temporary agency worker*	39%
	*Unemployed worker*	38%
	*Remaining worker*	23%

		

Missing values (range)		3.1%–7.8%

### Perceived health and RTW at 7–9 months after the start of sick leave

In table 2 the results are presented for perceived health and RTW. The most reported reason for absenteeism was having musculoskeletal complaints (34%) The perceived health (present, past and future) was in general poor. Only 18% of the workers reported that their present perceived health was good or very good and most of the workers experienced no change or even an aggravation of their health in the past 3 months (47% and 25% respectively). In addition, the majority of the workers were not hopeful with regard to their health in the near future. Finally, looking at RTW showed that 7–9 months after reporting sick, i.e. 4–6 months after inclusion/baseline, only 12% of the workers had completely returned to work and 7% had partially returned to work, whereas 81% had not (yet) started working again.

**Table 2:** Health variables and return-to-work measured at 7–9 months after the first day of reporting sick

**Variables**		**Cohort (n = 1077)**
**Health complaint**	*Cardio-vascular*	5%
	*Mental*	23%
	*Musculo-skeletal*	34%
	*Other*	24%
	*Combination of complaints*	14%

		

**Present perceived health**	*Very good*	3%
	*Good*	15%
	*Moderate*	31%
	*Fairly poor*	36%
	*Poor*	15%

		

**Perceived health in the past 3 months**	*Improved*	29%
	*Unchanged*	47%
	*Aggravated*	25%

		

**Health expectation in the coming 3 months**	*Will improve*	18%
	*No change*	31%
	*Will aggravate*	5%
	*Do not know*	46%

		

**Return-to-work (7–9 months after reporting sick)**	*Completely returned to work*	12%
	*Partly returned to work*	7%
	*Not returned to work*	81%

		

Missing values (range)		3.3%–3.8%

### Content and frequency of applied OHC interventions

In table 3 the content and frequency of the OHC interventions carried out by the insurance physicians of the SSA are presented. The most reported OHC intervention was 'the insurance physician discussed RTW' (49%; N = 528). On the other hand, 46% (N = 495) of the respondents reported not having discussed RTW with their insurance physician. The OHC intervention 'the insurance physician discussed actual starting with work' was reported by 28% (N = 302) of the workers, whereas 69% (N = 743) reported not having received this intervention. Even more striking was the reported number of the OHC intervention 'the insurance physician discussed and made a RTW action plan', which is mandatory according to the Dutch Gatekeeper Act. Only 19% (N = 205) of the respondents reported discussing and making of a RTW action plan by their insurance physician, while 74% (N = 797) of the workers reported that no RTW action plan was made. And finally, 'discussing training and/or education' and 'referral to a vocational rehabilitation agency' were also interventions reported only by a minority of the workers, respectively 13% (N = 140) and 17% (N = 183).

**Table 3:** Content and frequency of the occupational health care interventions carried out by the insurance physicians of the Social Security Agency

**Occupational health care interventions by the insurance physician of the SSA**		**Workers without an employment contract N = 1077**
Discussed RTW	*Yes*	49%
	*No*	46%
	*Do not know*	5%

		

Discussed training and/or education	*Yes*	13%
	*No*	83%
	*Do not know*	4%

		

Discussed actual starting with work again	*Yes*	28%
	*No*	69%
	*Do not know*	3%

		

Made and discussed RTW action plan	*Yes*	19%
	*No*	74%
	*Do not know*	7%

		

Referred to vocational rehabilitation agency	*Yes*	17%
	*No*	81%
	*Do not know*	2%

		

Missing values (range)		3.1%–4.6%

### OHC interventions as determinants for RTW

To examine if the reported OHC interventions were associated with RTW of the sick-listed workers without an employment contract, a logistic multiple regression analysis was conducted accounting for possible confounding variables and interaction effects. Confounding effects were found for type of worker, age, present perceived health, perceived health in the past 3 months, and health expectation in the coming 3 months. No interaction terms were included in the end model, since no important interaction effects were found. The results are presented in table 4. In the first part of the table, without adjusting for confounding variables, strong significant positive associations between RTW and reported OHC interventions were found for: 'OHC professional discussed RTW'; 'OHC professional discussed actual starting with work again'; and 'OHC professional made and discussed a RTW action plan'. A strong significant negative association with RTW was found for the intervention: 'OHC professional referred worker to a vocational rehabilitation agency'. In the second part of the table, after adjusting for confounding variables, a significant positive association with RTW remained for the OHC interventions: 'OHC professional discussed RTW'; and 'OHC professional made and discussed a RTW action plan'. The negative association with RTW, i.e. no RTW, for the intervention: 'OHC professional referred worker to a vocational rehabilitation agency' also remained significant. And finally, significant associations were found between RTW and the background variables: perceived health and age. Perceived good health was strongly associated with RTW (P = 0.000), whereas perceived bad health (p = 0.000) and age > 55 years (p = 0.021) were associated with no RTW.

**Table 4:** Associations between reported occupational health care interventions and return-to-work, not adjusted and adjusted for the measured baseline variables and health variables

Occupational health care intervention by the insurance physician	Association with RTW **not adjusted **for confounding variables*			Association with RTW **adjusted **for confounding variables*		
	
	*OR*	*95.0% CI for OR*	*p-value*	*OR*	*95.0% CI for OR*	*p-value*
*Discussed RTW*	1.644	1.142–2.368	0.008	1.573	1.030–2.404	0.036

*Discussed training and/or education*	0.899	0.529–1.529	0.694	0.829	0.451–1.525	0.547

*Discussed actual starting with work again*	1.982	1.387–2.833	0.000	1.003	0.659–1.526	0.990

*Made and discussed RTW action plan*	1.868	1.252–2.788	0.002	1.869	1.164–3.002	0.010

*Referred to vocational rehabilitation agency*	0.424	0.248–0.725	0.002	0.521	0.285–0.953	0.034

*confounding variables: type of worker; age; present perceived health; perceived health in the past 3 months; and health expectation in the coming 3 months

### Results of the non-response analysis

The sample of 3.500 persons was random taken from a population of 14.854 persons. On basis of the population data, provided by the SSA, we looked at the possibility of selectivity of the response (N = 1077). There were no important relative differences between the response data used in this study and the available population data as provided by the SSA. Therefore, we concluded that the non-response didn't harm the reliability of the data used in this study.

## Discussion

The aim of this cohort study was to examine characteristics of workers without an employment contract, sick-listed for at least 13 weeks; to examine OHC for this group of sick-listed workers; and to examine the association between applied OHC interventions and RTW. The sick-listed workers without an employment contract in this study were characterised by a low level of education. At 7–9 months after the first day of reporting sick most of the workers viewed their (present, past and future) health as fairly poor or poor and the most reported reason for absenteeism was having musculoskeletal complaints. Only 19% of the workers without an employment contract had (partially or completely) returned to work, whereas the majority (81%) of the workers had not (yet) started working again. When looking at the reported OHC interventions, the most frequently reported (49%) intervention was 'the OHC professional discussed RTW'. However, the intervention 'the OHC professional discussed and made a RTW action plan', which is mandatory according to the Dutch legislation for OHC, was reported by only 19% of the workers while 74% of the workers reported that no RTW action plan was made by their insurance physician. Finally, a logistic multiple regression analysis showed a significant positive association between RTW and the reported interventions: 'OHC professional discussed RTW'; and 'OHC professional made and discussed a RTW action plan'. In addition, a significant negative association with RTW, i.e. no RTW, was found for the intervention: 'OHC professional referred worker to a vocational rehabilitation agency'.

### RTW of sick-listed workers without an employment contract

After 7–9 months only 19% of the sick-listed workers without an employment contract had partially (7%) or completely (12%) returned to work, whereas the majority of the workers had not (yet) returned to work. A comparable TNO study among sick-listed employees[16] showed 7–9 months after reporting sick a RTW rate of 81% (31% partially and 50% completely). With the remark that other study designs are needed to further investigate this considerable difference in RTW rate, two possible explanations for this phenomenon will be discussed. First, as mentioned earlier, these workers represent a vulnerable group within the working population with a greater distance to the labour market[2,13,15]. Finding a workplace and getting an employment contract is therefore in any case more difficult for these workers. It is also likely that being sick-listed adds to this already present 'labour market handicap'. This is supported by findings in international literature [18-20], indicating that the work status before sickness absence is a prognostic factor for the duration of sick leave and work disability. The presence of a workplace/employer to return to seems to be an important factor in the success of RTW (Vermeulen et al., 2009, submitted). Secondly, an important finding of this study is the relatively low amount of received OHC interventions as reported by the respondents. These interventions are obligatory according to Dutch legislation for OHC and in line with this higher numbers could be expected. In this study all respondents were at least 13 weeks sick-listed and should have been invited to visit the insurance physician at least once. However, summoning to consulting hours was reported by only 54% of the respondents. Therefore, a low rate of visits to the insurance physician appears to be an explanation for the low number of OHC interventions. On the other hand, an important factor also seems to be insufficient OHC practise by the professionals of the SSA. Obligatory interventions, such as making of a RTW action plan, and discussing actual starting with work again, were reported by only 19% and 28% of the respondents respectively. If a low rate of visits to the insurance physician would be the main reason for the low number of applied OHC interventions, the number of reported obligatory interventions should be closer to the found rate for visiting the insurance physician.

### Association between RTW and received occupational health care interventions

The logistic multiple regression analysis showed that the interventions 'OHC professional discussed RTW' and 'OHC professional made and discussed a RTW action plan' were positively associated with RTW. In addition, a striking finding was the strong significant positive association found for RTW and the OHC intervention 'discussing actual starting with work again', which disappeared when adjusted for confounding variables. Further examination of the results showed a strong association between the intervention 'OHC professional discussed actual starting with work again' and the (present) perceived health status, i.e. perceiving health as good. Therefore, it is likely that experiencing a good and/or improved health, as part of the recovery process, resulted in talking about actual starting with work again (initiated by either the worker or the insurance physician of the SSA) and eventually actual RTW.

### Meaning of study findings in an international perspective

Workers with flexible labour market arrangements work in more hazardous psychological and physical work environments (painful or tiring position, intense noise, repetitive tasks) than employees[2], with higher hazard exposures, disease risk and injury rates[11]. International literature also reports higher rates of mortality among temporary employment and unemployment [21-25]. In addition, as mentioned above, this vulnerable working population is characterised by a greater distance to the labour market[2,13,26].

However, there are many countries where workers without an employment contract, i.e. with flexible work arrangements, have no or only limited access to vocational rehabilitation interventions [27-29]. From this perspective, the frequency of reported OHC interventions found in this Dutch study, can even be considered as high.

Looking at reviews concerning occupational health interventions and RTW shows that most studies are aimed at 1. identifying prognostic factors regarding RTW [30-32]; 2. assessing the effectiveness of OHC intervention programs [33-41]; and 3. identifying the effective components of OHC intervention programs[32,42-44]. Many of these OHC intervention programs are workplace-based or at least contain a workplace component. Also, literature suggests that employer participation, a supportive work climate, cooperation between labour and management, and work accommodations are important factors in facilitating return-to-work[32,44]. However, a major obstacle for the sick-listed worker without an employment contract is the absence of a workplace to return to. In international literature the absence of adequate OHC for the vulnerable workers without an employment contract or with a flexible labour agreement is a rarely described problem. However, it can be expected that this problem will only increase because the trend towards more flexible labour market arrangements is growing in West-European countries[1,2]. In our opinion, this study contributes to knowledge, i.e. insight into current OHC practise, needed for the development of adequate, i.e. tailor-made, OHC to optimize vocational rehabilitation and RTW of the vulnerable workers with flexible labour agreements.

Furthermore, the attention paid in this study to the vulnerable working population, is also in line with the goals of the World Health Organisation (WHO), which aims at 'OHC for all' and a change of focus from occupational health to workers health.

### Strengths

Strength of this study is its large sample size. It is the first time, that characteristics of a large cohort of sick-listed workers without an employment contract are described, in particular the amount of reported OHC interventions, and actual RTW. Another strength of this study is the focus on a vulnerable group within the working population, i.e. workers without an employment contract. In the international literature this subject is rarely described in spite of the extent of the problem; by definition, RTW will always be more difficult since sick-listed workers without an employment contract have (in most cases) no workplace/employer to return to.

### Weaknesses

The first limitation of this study is the fact that all findings are based on self-reported data. Therefore, the presence of recall-bias may have influenced the findings in this study. It is possible that the respondents who had already successfully (partially or completely) returned to work, i.e. only 19% in this study, remembered more OHC interventions, resulting in an overestimation of the associations between the reported interventions and RTW. On the other hand, due to the low RTW rate, a lot of the respondents had more opportunities to receive OHC interventions.

A second limitation is the possibility of a wrong estimation of the amount of applied OHC interventions due to the fairly high number of non-responders. However, we found no indication for this in the non-response analysis.

And finally, the aim of this study was to describe the content and frequency of applied OHC interventions and to examine the association between these interventions and RTW. The causal nature of the associations found between RTW and applied OHC interventions in this study needs to be investigated in future research.

### Research challenges for present and future

Given the fact that in this study only 19% of the sick-listed workers without an employment contract had (partially or completely) returned to work 7–9 months after the first day of reporting sick, there can be gained a lot by efforts reducing short- and long-term sickness absence and work disability of these vulnerable workers[26]. A potentially useful RTW intervention for sick-listed workers without an employment can be e.g. the presence of a therapeutic workplace to return to. Because different stakeholders are involved[45] and centralized coordination of RTW of the sick-listed worker is essential[44], realizing structural collaboration and communication between all stakeholders involved should be an important part of such an intervention. Currently, based on the Intervention Mapping (IM) process [46-48], a participatory RTW intervention was developed for workers without an employment contract sick-listed due to musculoskeletal disorders (Vermeulen et al., 2009, submitted). Tailoring of an RTW intervention to a specific target group with IM proved also to be successful in other OHC research[49]. The new intervention is based on a previous developed and successful participatory intervention for employees sick-listed due to low back pain[50,51] and will be evaluated in an randomised control trial in the eastern part of the Netherlands. To study the effect of a structured stepwise program for realizing a RTW implementation plan and creating an actual therapeutic workplace as stepping stone to permanent RTW.

## Conclusion

It is the first time, that characteristics of a large cohort of sick-listed workers without an employment contract are described, in particular concerning the content and frequency of applied OHC interventions, RTW and the relationship between these. To explore the causal nature of these associations, an experimental or prospective study is needed for the vulnerable working population, i.e. workers without an employment contract. This should include further research for the development of tailor-made OHC interventions to optimize the frequency and content of these interventions and to evaluate the effect of these interventions on RTW of the vulnerable workers.

## Competing interests

The authors declare that they have no competing interests.

## Authors' contributions

JFY developed the study design and was responsible for data collection. SJV and AJMS conducted the analyses. SJV, SJT, AJMS, and JRA drafted the manuscript. JRA, JFY and SJV act as guarantors of the study. All authors read and approved the final manuscript.

## Pre-publication history

The pre-publication history for this paper can be accessed here:



## References

[B1] Benavides FG, Benach J, Diez-Roux AV, Roman C (2000). How do types of employment relate to health indicators? Findings from the Second European Survey on Working Conditions. J Epidemiol Community Health.

[B2] Benach J, Amable M, Muntander C, Benavides FG (2002). The consequences of flexible work for health: are we looking at the right place?. J Epidemiol Community Health.

[B3] Benach J, Gimeno D, Benavides FG, Martínez JM, Del Mar Torné M (2004). Types of employment and health in the European Union: changes from 1995 to 2000. European Journal of Public Health.

[B4] Benach J, Muntaner C (2007). Precarious employment and health: developing a research agenda. J Epidemiol Community Health.

[B5] Benach J, Benavides FG, Platt S, Diez-Roux A, Muntaner C (2000). The health-damaging potential of new types of flexible employment: a challenge for public health researchers. Am J Public Health.

[B6] Jin RL, Shah CP, Svoboda TJ (1995). The impact of unemployment on health: a review of the evidence. CMAJ.

[B7] Dooley D, Fielding J, Levi L (1996). Health and unemployment. Annu Rev Public Health.

[B8] Virtanen M, Kivimäki M, Elovainio M, Vahtera J (2002). Selection from fixed term to permanent employment: prospective study on health, job satisfaction, and behavioural risks. J Epidemiol Community Health.

[B9] Virtanen P, Liukkonen V, Vahtera J, Kivimäki M, Koskenvuo M (2003). Health inequalities in the workforce: the labour market core-periphery structure. Int J Epidemiol.

[B10] Roos E, Lahelma E, Saastamoinen P, Elstad JI (2005). The association of employment status and family status with health among women and men in four Nordic countries. Scand J Public Health.

[B11] Quinlan M, Mayhew C, Bohle P (2001). The global expansion of precarious employment, work disorganization, and consequences for occupational health: a review of recent research. Int J Health Serv.

[B12] Centraal bureau voor de statistiek [Statisticus Netherlands]. Cijfers arbeid en sociale zekerheid: ziekteverzuim, arbeidsongeschiktheid, uitkeringen sociale zekerheid [Labour and social security figures: sickness absence, work disability and disability pensions]. http://www.cbs.nl/en-GB/menu/cijfers.

[B13] Arents MR, Dorenbos I, Vogelaar B, Vrijhof B, Landheer W (2003). Aard en oorzaken ziekteverzuim Uitzendbranche [Nature and causes sickness absence among temporary agency workers].

[B14] Uitvoeringsinstituut Werknemersverzekeringen [Dutch Institute for Employee Benefit Schemes] (2005). Instroomcijfers WAO 2004 [Awarded disability pensions figures 2004].

[B15] Veerman TJ (2005). Vroegtijdige reïntegratie uitzendkrachten [Early return-to-work of temporary agency workers].

[B16] Ybema JF, Lagerveld S, Berg R Van den (2004). Rapport werking Wet verbetering Poortwachter onder vangnetters – DEEL 1: eerste cohort [Report working Improved Gatekeeper Law among non-employees – PART 1: first cohort].

[B17] Christelijk Nationaal Vakverbond (CNV) [Christan National Union] (2001). Tijd voor reïntegratie: onderzoek onder langdurig zieke werknemers naar de relatie tussen reïntegratieactiviteiten en het moment van WAO beoordeling [It's time for vocational rehabilitation: a study on the relationship between vocational rehabilitation activities for long term sick-listed employees and the moment of assessment for a long term disability pension].

[B18] Abásolo L, Carmona L, Lajas C, Candelas G, Blanco M, Loza E, Hernández-García, Jover JA (2008). Prognostic factors in short-term disability due to musculoskeletal disorders. Arthritis Rheum.

[B19] Cheadle A, Franklin G, Wolfhagen C, Savarino J, Liu PY, Salley C, Weaver M (1994). Factors influencing the duration of work-related disability: a population-based study of Washington State workers' compensation. Am J Public Health.

[B20] Bartley M, Sacker A, Clarke P (2004). Employment status, employment conditions, and limiting illness: prospective evidence from the British household panel survey 1991–2001. J Epidemiol Community Health.

[B21] Kivimäki M, Vahtera J, Virtanen M, Elovainio M, Pentti J, Ferrie JE (2003). Temporary employment and risk of overall and cause-specific mortality. Am J Epidemiol.

[B22] Hirokawa K, Tsutusmi A, Kayaba K (2006). Impacts of educational level and employment status on mortality for Japanese women and men: the Jichi Medical School cohort study. Eur J Epidemiol.

[B23] Nylén L, Voss M, Floderus B (2001). Mortality among women and men relative to unemployment, part time work, overtime work, and extra work: a study based on data from the Swedish twin registry. Occup Environ Med.

[B24] Voss M, Nylén L, Floderus B, Diderichsen F, Terry PD (2004). Unemployment and early cause-specific mortality: a study based on the Swedish twin registry. Am J Public Health.

[B25] Ahs AM, Westerling R (2006). Mortality in relation to employment status during different levels of unemployment. Scand J Public Health.

[B26] Reijenga FA, Veerman TJ, Berg N van den (2006). Onderzoek evaluatie wet verbetering poortwachter [Evaluation of the Improved Gatekeeper Act].

[B27] Ahs AM, Westerling R (2006). Health care utilization among persons who are unemployed or outside the labour force. Health Policy.

[B28] Virtanen P, Kivimäki M, Vahtera J, Koskenvuo M (2006). Employment status and differences in the one-year coverage of physician visits: different needs or unequal access to services?. BMC Health Serv Res.

[B29] Watson PJ, Booker CK, Moores L, Main CJ (2004). Returning the chronically unemployed with low back pain to employment. Eur J Pain.

[B30] Shaw WS, Pransky G, Fitzgerald TE (2001). Early prognosis for low back disability: intervention strategies for health care providers. Disabil Rehabil.

[B31] Peters J, Pickvance S, Wilford J, Macdonald E, Blank L (2007). Predictors of delayed return to work or job loss with respiratory ill-health: a systematic review. J Occup Rehabil.

[B32] Zampolini M, Bernardinello M, Tesio L (2007). RTW in back conditions. Disabil Rehabil.

[B33] Frank J, Sinclair S, Hogg-Johnson S, Shannon H, Bombardier C, Beaton D, Cole D (1998). Preventing disability from work-related low-back pain. New evidence gives new hope-if we can just get all the players onside. CMAJ.

[B34] Verbeek JH (2001). Vocational rehabilitation for workers with back pain. Scand J Work Environ Health.

[B35] Weir R, Nielson WR (2001). Interventions for disability management. Clin J Pain.

[B36] Franche RL, Cullen K, Clarke J, Irvin E, Sinclair S, Frank J, The Institute for Work & Health (IWH) Workplace-Based RTW Intervention Literature Review Research Team (2005). Workplace-based return-to-work interventions: a systematic review of the quantitative literature. J Occup Rehabil.

[B37] Hlobil H, Staal JB, Spoelstra M, Ariëns GA, Smid T, van Mechelen W (2005). Effectiveness of a return-to-work intervention for subacute low-back pain. Scand J Work Environ Health.

[B38] Ruotsalainen JH, Verbeek JH, Salmi JA, Jauhiainen M, Laamanen I, Pasternack I, Husman K (2006). Evidence on the effectiveness of occupational health interventions. Am J Ind Med.

[B39] Williams RM, Westmorland MG, Lin CA, Schmuck G, Creen M (2007). Effectiveness of workplace rehabilitation interventions in the treatment of work-related low back pain: a systematic review. Disabil Rehabil.

[B40] Tompa E, de Oliveira C, Dolinschi R, Irvin E (2008). A systematic review of disability management interventions with economic evaluations. J Occup Rehabil.

[B41] van Oostrom SH, Driessen MT, de Vet HC, Franche RL, Schonstein E, Loisel P, van Mechelen W, Anema JR (2009). Workplace interventions for preventing work disability. Cochrane Database Syst Rev.

[B42] Williams RM, Westmorland M (2002). Perspectives on workplace disability management: a review of the literature. Work.

[B43] Durand MJ, Vézina N, Loisel P, Baril R, Richard MC, Diallo B (2007). Workplace interventions for workers with musculoskeletal disabilities: a descriptive review of content. J Occup Rehabil.

[B44] Briand C, Durand MJ, St-Arnaud L, Corbière M (2008). How well do return-to-work interventions for musculoskeletal conditions address the multicausality of work disability?. J Occup Rehabil.

[B45] Nederlandse Vereniging voor Verzekeringsgeneeskunde [Dutch Association of Insurance Medicine] (2005). Arborol: verslag werkgroep arborol van de NVVG [Occupational health care by the Social Security Agency: report of a study group of the Dutch Association of Insurance Medicine].

[B46] Bartholomew LK, Parcel GS, Kok G (1998). Intervention mapping: a process for developing theory- and evidence-based health education programs. Health Educ Behav.

[B47] Bartholomew LK, Parcel GS, Kok GJ, Gottlieb NH (2001). Intervention Mapping: designing theory and evidence-based health promotion programs.

[B48] Bartholomew LK, Parcel GS, Kok G, Gottlieb NH (2006). Planning health promotion programs: an Intervention Mappping approach.

[B49] van Oostrom SH, Anema JR, Terluin B, Venema A, de Vet HC, van Mechelen W (2007). Development of a workplace intervention for sick-listed employees with stress-related mental disorders: Intervention Mapping as a useful tool. BMC Health Serv Res.

[B50] Anema JR, Steenstra IA, Urlings IJ, Bongers PM, De Vroome EM, van Mechelen W (2003). Participatory ergonomics as a return-to-work intervention: a future challenge?. Am J Ind Med.

[B51] Anema JR, Steenstra IA, Bongers PM, de Vet HC, Knol DL, Loisel P, van Mechelen W (2007). Multidisciplinary rehabilitation for subacute low back pain: graded activity or workplace intervention or both? A randomized controlled trial. Spine.

